# ZMK1 Is Involved in K^+^ Uptake and Regulated by Protein Kinase ZmCIPK23 in *Zea mays*

**DOI:** 10.3389/fpls.2021.517742

**Published:** 2021-03-03

**Authors:** Wu Han, Yun Ji, Wei Wu, Jin-Kui Cheng, Han-Qian Feng, Yi Wang

**Affiliations:** ^1^State Key Laboratory of Plant Physiology and Biochemistry (SKLPPB), College of Biological Sciences, China Agricultural University, Beijing, China; ^2^State Key Laboratory for Managing Biotic and Chemical Threats to the Quality and Safety of Agro‐products, Institute of Virology and Biotechnology, Zhejiang Academy of Agricultural Sciences, Hangzhou, China; ^3^Center for Crop Functional Genomics and Molecular Breeding, China Agricultural University, Beijing, China

**Keywords:** *Zea mays*, ZmCIPK23, K^+^ uptake, K^+^ channel, ZMK1

## Abstract

Potassium (K^+^) is one of essential mineral elements for plant growth and development. K^+^ channels, especially AKT1-like channels, play crucial roles in K^+^ uptake in plant roots. Maize is one of important crops; however, the K^+^ uptake mechanism in maize is little known. Here, we report the physiological functions of K^+^ channel ZMK1 in K^+^ uptake and homeostasis in maize. *ZMK1* is a homolog of *Arabidopsis AKT1* channel in maize, and mainly expressed in maize root. Yeast complementation experiments and electrophysiological characterization in *Xenopus* oocytes indicated that ZMK1 could mediate K^+^ uptake. *ZMK1* rescued the low-K^+^-sensitive phenotype of *akt1* mutant and enhanced K^+^ uptake in *Arabidopsis*. Overexpression of *ZMK1* also significantly increased K^+^ uptake activity in maize, but led to an oversensitive phenotype. Similar to AKT1 regulation, the protein kinase ZmCIPK23 interacted with ZMK1 and phosphorylated the cytosolic region of ZMK1, activating ZMK1-mediated K^+^ uptake. *ZmCIPK23* could also complement the low-K^+^-sensitive phenotype of *Arabidopsis cipk23*/*lks1* mutant. These findings demonstrate that ZMK1 together with ZmCIPK23 plays important roles in K^+^ uptake and homeostasis in maize.

## Introduction

Potassium (K^+^) is an essential macronutrient used by plants. It plays important roles in enzyme activation, stomata movement, osmotic regulation, and plasma membrane potential control (Clarkson and Hanson, 1980; Leigh and Jones, 1984; Holdaway-Clarke and Hepler, 2003). The normal cytosolic K^+^ concentration is within 20–100 mM range (Leigh and Jones, 1984). However, the typical K^+^ concentration in the soil varies between 0.1 and 1 mM (Maathuis, 2009). Potassium is important for crop yield and quality. Potassium deficiency is one of limiting factors for agricultural production, especially in Asia^1^ (Chen et al., 2008; Römheld and Kirkby, 2010). There exists uneven distribution of potassium in the world. The price of potassium has been increasing year by year. China’s potassium production is relatively limited, 50% of potassium usage depends on importation (Tan et al., 2017). It can be seen that soil potassium deficiency and potash shortage have become a main obstacle, which limits the development of China agricultural production. Therefore, the absorption of K^+^ from the environment to the plants is crucial for K^+^ utilization efficiency.

Previous studies show that K^+^ absorption and translocation is mainly meditated by a group of channels and transporters. Plant roots have evolved two-phase mechanisms for K^+^ absorption (Epstein et al., 1963). In *Arabidopsis*, AKT1 channel and HAK5 transporter are considered as the two main systems for K^+^ uptake, especially when the environmental K^+^ concentration is below 100 μM. The *hak5 akt1* double mutants fail to grow on medium containing up to 100 mM K^+^ (Hirsch et al., 1998; Rubio et al., 2000; Gierth et al., 2005; Pyo et al., 2010). Recently, the K^+^ transporters ZmHAK5 and ZmHAK1 were reported to play important roles in K^+^ uptake and distribution in maize under low K^+^ (LK) stress (Qin et al., 2019). AKT1 has been identified as an inward-rectifying K^+^ channel participating in K^+^ uptake in *Arabidopsis* root cells (Lagarde et al., 1996; Hirsch et al., 1998). Loss-of-function mutant of *AKT1* displays ammonium dependent sensitive phenotype under low K^+^ conditions (Hirsch et al., 1998; Xu et al., 2006). AKT1 is electrically silent when expressed in *Xenopus* oocytes, thus it has been presumed that potential regulating factors may be needed to activate its activity. Studies showed that activation of AKT1 requires the interaction with the CBL-interacting protein kinase23 (CIPK23) and Calcineurin B-Like protein1/9 (CBL1/9; Xu et al., 2006; Lee et al., 2007). Another Calcineurin B-Like protein CBL10 was identified to negatively regulate the activity of AKT1. *CBL10* overexpressing lines showed similar sensitive phenotype to *akt1* mutant under low potassium conditions, suggesting that the activity of AKT1 is tightly controlled in plants (Ren et al., 2013).

Many *AKT1* homolog genes have been identified in other plant species. *OsAKT1*, an homolog in rice (*Oryza sativa*), was functionally expressed in HEK293 cells, and showed high ion uptake specificity to K^+^ (Fuchs et al., 2005). OsAKT1 is critical for K^+^ uptake in rice and modulated by the rice CBL1-CIPK23 complex (Li et al., 2014). *ZMK1*, an homolog in maize (*Zea mays*), showed auxin-induced expression pattern and was presumed to participate in coleoptile growth (Philippar et al., 1999). In Grapevine (*Vitis vinifera*), two AKT1-like K^+^ channels have been identified, VvK1.1 and VvK1.2, both were electrically silent when expressed alone in *Xenopus* oocytes. Like AKT1, VvK1.1 is mainly expressed in cortical cells, and can be activated by *Arabidopsis* CBL1/CIPK23 complex (Cuéllar et al., 2010, 2013). The transcript of *VvK1.2* is predominantly accumulated in maturing berries. When co-expressed with the pairs VvCIPK3-VvCBL2 or VvCIPK4-VvCBL1, VvK1.2 can mediate K^+^ currents (Cuéllar et al., 2013).

Maize is an important food and commercial crop. It represents one of the most widely grown crops worldwide, which is crucial for the global food security. The cultivation and growth of maize and the grain yield are greatly affected by the availability of potassium in the soil (Pettigrew, 2008). Genetic improvement of maize K^+^ utilization efficiency is an important strategy for solving this problem. Previously, only a few AKT1-like potassium channels have been cloned in maize. The physiological functions and regulation mechanisms of these channels have not been characterized in detail. In this study, we show that K^+^ channel ZMK1 plays important roles in K^+^ uptake and homeostasis in maize. Similar to AKT1, ZMK1 is activated by the ZmCBL1-ZmCIPK23 protein kinase complex.

## Materials And Methods

### Plant Materials

The *Arabidopsis* (*Arabidopsis thaliana*) ecotype Columbia was used as wild type throughout this study. The *akt1* and *cipk23* were obtained as previously described (Xu et al., 2006). The maize ZMK1 and ZmCIPK23 coding sequence were cloned into the pCAMBIA1300 vector driven by the ubiquitin promoter. The ubiquitin promoter is a constitutive promoter. The *pCAMBIA1300* vectors were transformed to *akt1* and *cipk23, respectively,* to obtain the transgenic lines. The background of *akt1* and *cipk23* is col-0. Transformation of *Arabidopsis* was performed with *Agrobacterium* (strain GV3101) by the floral dip method (Clough and Bent, 1998).

### Ethics Statement

Animal studies were conducted in accordance with the ethical guidelines of Ministry of Agriculture (Beijing, China). All procedures were approved by the Animal Care ethics Committee of China Agricultural University.

### Plant Growth Conditions and Phenotypic Observations

The *Arabidopsis* seeds were surface sterilized using 6% (v/v) NaClO and rinsed with distilled water for five times. After cold stratification at 4°C dark chamber for 3 days, the seeds were germinated on Murashige and Skoog (MS) medium [with 0.8% (w/v) agar and 3% (w/v) sucrose] under constant illumination at 60 mol m^−2^ s^−1^. For low K^+^ treatment assay, 4-day-old seedlings grown on MS medium were transferred to MS (20 mmol/L K^+^) medium or LK (100 μmol/L K^+^) medium for another 7 days. The LK medium was prepared by substituting KNO_3_ and KH_2_PO_4_ in MS medium with NH_4_NO_3_ and NH_4_H_2_PO_4_, respectively. The final K^+^ concentration was adjusted to 100 μM by adding KCl.

Seedlings were transferred to different media for 7 or 10 days, and then photographs were taken. The 7-day-old seedlings grown on different media were harvested. The dry weight was measured as biomass. In addition, the fresh shoots of 7-days-old seedlings were harvested for chlorophyll content measurement. The chlorophyll was extracted in 80% acetone (v/v) in the dark for 2 days. Then, the absorbance of the extraction buffer at 645 and 663 nm was measured. Each biological replicate contained more than 15 individual plants.

The hydroponic system was used to culture maize. After the seeds were disinfected, the vermiculite was germinated for 6 days. The endosperm was removed and transferred to high K^+^ (1.85 mmol/L) for 2 days. Then the seedlings were transferred to high K^+^ (HK, 1.85 mmol/L) and low K^+^ (0.03 mmol/L) hydroponic solution. The complete nutrient solution (HK) was modified Hoagland solution (Arnon and Hoagland, 1939) consisting of 0.75 mmol/L K_2_SO_4_, 2 mmol/L CaNO_3_•4H_2_O, 0.65 mmol/L MgSO_4_•7H_2_O, 0.1 mmol/L KCl, 0.25 mmol/L KH_2_PO_4_, 0.1 mmol/L Fe-EDTA, and micronutrients (1 μmol/L MnSO_4_, 1 μmol/L ZnSO_4_•7H_2_O, 0.01 μmol/L CuSO_4_•5H_2_O, 0.005 μmol/L (NH_4_)_6_Mo_7_O_24_•4H_2_O, and 1 μmol/L H_3_BO_3_), pH 6.0. For hydroponic culture under low K^+^ conditions, the K_2_SO_4_ was reduced and replaced with NaH_2_PO_4_•2H_2_O. The plant materials cultured in a greenhouse with a 14-h light (28°C)/10-h dark (26°C).

### Field Cultivation of Maize

Large-scale field season in 2016–2017 at China Agricultural University’s experimental stations (Zhuozhou, Hebei Province). The normal K supply for maize cultivation in most areas of China is about 1.8 kg K/100 m^2^. Thus, we did not use potash in Zhuozhou for low-K conditions and 1.8 kg K/100 m^2^ (using K_2_SO_4_ as the major K source) for high-K conditions. P_2_O_5_ was used as phosphorus fertilizer (4.8 kg P/100 m^2^). Urea was used as nitrogen fertilizer (6.0 kg N/100 m^2^). The spacing between plants was 25 cm.

### K^+^ Content Measurement

For K^+^ content measurement in *Arabidopsis*, 4-day-old *Arabidopsis* seedlings grown in MS medium were transferred to LK or MS medium and treated for 7 days. The shoots and roots were harvested separately. Fifty or one hundred seedlings were collected as one biological replicate sample for plants grown on MS or LK medium, respectively. Data were obtained from three biological replicates.

The collected samples were dried at 80°C for 24 h to a constant weight and then the dry weight was measured. The samples were treated in a muffle furnace at 300°C for 1 h, and then 575°C for 5 h. The ashes were dissolved and diluted in 0.1 N HCl. The K^+^ concentrations were measured using the 4100-MP AES device (Agilent).

Uniform maize seedlings with two fully expanded leaves were grown in LK (0.03 mmol/L K^+^ or 0.05 mmol/L K^+^) or HK^+^ (1.85 mmol/L K^+^) nutrient solution for 18 or 7 days; the nutrient solution was replaced every 2 days. Five plants were collected as one biological replicate sample. Data were obtained from five biological replicates. The shoots and roots were collected separately in craft paper bags and dried in an 80°C oven. When the samples were dried, dry weight was measured with an electronic balance (0.0001 accuracy). After grinding the material, we weigh 0.2 g samples into the crucibles. Then, the crucibles were incinerated in a muffle furnace at 300°C for 3 h and 575°C for 6 h. Add 0.1 N HCl to the ashed material for dissolution. The K^+^ contents of the solutions were measured using a 4100-MP AES spectrometer (Agilent).

### Yeast Complementation Assay

The CDS of *ZMK1* was cloned in the p416-GPD vector. Empty p416-GPD vector, was transformed into yeast strain R5421 lacking two endogenous K^+^ transporter genes. Yeast strain R5421 was used as negative control and yeast strain R757 was used as a positive control (Gaber et al., 1988; Nakamura et al., 1997). The genotype of yeast strains R5421 is MAT*a ura3-52 leu2 trk1∆his3∆200his4-15 trk2∆pCK64.* Monoclonal yeast cells were cultured in YPDA medium containing 100 mmol/L KCl at 30°C until the OD_600_ reached 0.8. After centrifugation at 6000 *g* for 1 min and three washes with sterile double-distilled water, the cells were resuspended in sterile double-distilled water to OD_600_ 0.8. The yeast cells were serially diluted 10-fold and grow on different K^+^ concentrations of AP medium for 4 days.

### Transcriptional Level Quantification

For detection of gene tissue expression in maize, the maize B73-329 seeds were grown to the three leaves stage and the tissue samples were cut with a blade and frozen in liquid nitrogen. Each sample was obtained from seedlings.

For LK treatment, maize seedlings grown to the V3 in 1 mM K^+^ were transferred to hydroponic solution containing 1 or 0 mM K^+^ respectively for 12 or 24 h. The root tissues were quickly frozen in liquid nitrogen.

Total RNA was extracted from plants using Trizol reagent (Invitrogen, http://www.invitrogen.com). For northern blot assay, 20 g total RNA was separated with 1.2% denaturing agarose gels containing 2% formaldehyde and transferred Hybond-N^+^ membranes. Membranes were heated at 80°C for 2 h and cross-linked by UV. Hybridization was performed overnight at 65°C with [*α*-^32^P]CTP-labeled DNA oligonucleotides in hybridization buffer. All labeling was carried out using Random Prime Labeling System from Amersham Biosciences. The blots were washed sequentially with 2 × SSC + 0.5% (w/v) SDS, 1 × SSC + 0.5% (w/v) SDS, and 0.5 × SSC + 0.5% (w/v) SDS. Washed blots were exposed to X-ray films.

For quantitative reverse transcription PCR (RT-qPCR) analysis, total RNA were digested with DNase I to remove genomic DNA. cDNA was synthesized from 2 g treated RNA using SuperScript II RNase H reverse transcriptase (Invitrogen) with random hexamer primers. Quantitative real-time PCR was performed with gene specific primer using Power SYBR Green PCR Master Mix (Applied Biosystems). Relative gene expression was calculated with 2-^*Δ*ΔCT^ method by normalization to *ZmGAPDH* for semi-quantitative RT-PCT analysis. Data were obtained from three biological replicates.

### Electrophysiological Measurement in *Xenopus* Oocytes

The CDS for *ZMK1* (GRMZM2G171279), *ZmCIPK23* (GRMZM2G013236), *ZmCBL1* (GRMZM2G107575), and *ZmCBL9* (GRMZM2G015324) was cloned into the pGEMHE vector. The plasmids were linearized and *in vitro* transcription of cRNAs was performed with T7 RiboMAX™ large-scale RNA production system (Promega) according to manufacturer’s instructions. Oocytes were obtained from *Xenopus laevis* and RNAs were mixed and injected. The ZMK1 expressed oocytes were injected with 6 ng of ZMK1 copy cRNA (in 50 nl). The ZmCBL1 (or ZmCBL9), ZmCIPK23, and ZMK1 co-expressed oocytes were injected with cRNA mixture of ZmCBL1 (or ZmCBL9), ZmCIPK23, and ZMK1 (0.6:0.6:6 ng in 50 nl). The injected oocytes were cultured at 17°C in the modified Bath’s solution for 36 h. Two-electrode voltage-clamp measurements on oocytes were performed as previously described (Ren et al., 2013). Quantitative data were obtained from at least five oocytes.

### Yeast Two-Hybrid Assays

The coding sequences for *ZmCBL1*, *ZmCBL9* and the cytosolic C-terminus region of *ZMK1* were cloned into the pGADT7 vector and the coding sequence for *ZmCIPK23* was cloned into pGBKT7 vector. The vectors were transformed into yeast strain AH109 and the positive clones were selected SC solid medium lacking Leu and Trp. Clones were incubated in SC liquid medium lacking Leu and Trp at 28°C to an absorbance of 1.0 at 600 nm. Aliquots (5 μl) of serial dilutions were spotted on selective medium (lacking Leu, Trp, His, and Ade) and non-selective medium (lacking Leu, and Trp).

### *In vitro* Phosphorylation Assay

The *in vitro* kinase assay was performed according to previously described method with small modification (Hashimoto et al., 2012). Briefly, StrepII-tagged ZmCBL1, ZmCBL9, and ZmCIPK23 proteins were generated using a RTS 100 wheat germ CECF kit and purified with Strep-Tactin Macroprep (IBA). GST tagged ZMK1-C protein was expressed in *E. coli*. and purified with Glutathione Sepharose 4B (GE Healthcare). About 1 μg ZMK1-C protein and 0.2 μg ZmCBL1, ZmCBL9, and ZmCIPK23 proteins were used in the phosphorylation reaction. Purified proteins were incubated for 30 min at 30°C in 30 μl reactions containing 20 mM Tris (pH 8.0), 1 mm MnSO_4_, 0.5 mM CaCl_2_, 1 mM DTT, 10 μM ATP, and 3 μCi of [*γ*-32P]ATP. The reactions were terminated by adding 6 μl 6×SDS protein sampling buffer and then subjected to SDS-PAGE. SDS gels were stained with Coomassie blue, and radioactively labeled proteins were visualized by autoradiography.

### Kinetic Analysis of K^+^ Uptake

Sterilize the maize seeds with 10% H_2_O_2_ for 30 min and plant them in a box with a meteorite. The seedlings, in which the endosperm was removed, were hydroponically cultured to two fully expanded leaves and transferred to starvation treatment for 24 h (containing 2 mmol/L CaNO_3_•4H_2_O, 1.4 mmol/L MgSO_4_•7H_2_O, 0.25 mmol/L NaH_2_PO_4_•2H_2_O, 0.1 mmol/L Fe-EDTA, and micronutrients (pH 5.9). For K^+^-depletion experiments, seedlings were transferred to depletion treatment (containing 2 mmol/L CaNO_3_•4H_2_O, 1.4 mmol/L MgSO_4_•7H_2_O, 0.25 mmol/L K_2_PO_4_•2H_2_O, 0.1 mmol/L NaCl, 0.1 mmol/L Fe-EDTA, pH 5.9). The depletion solutions were sampled at different time points as indicated. The K^+^ contents of the solutions were measured using a 4100-MP AES spectronmeter (Agilent). The data were fitted to sigmoidal curves. Data were obtained from three biological replicates.

## Results

### Phylogenetic Analyses of Shaker Potassium Channels in Maize

**Figure 1:**
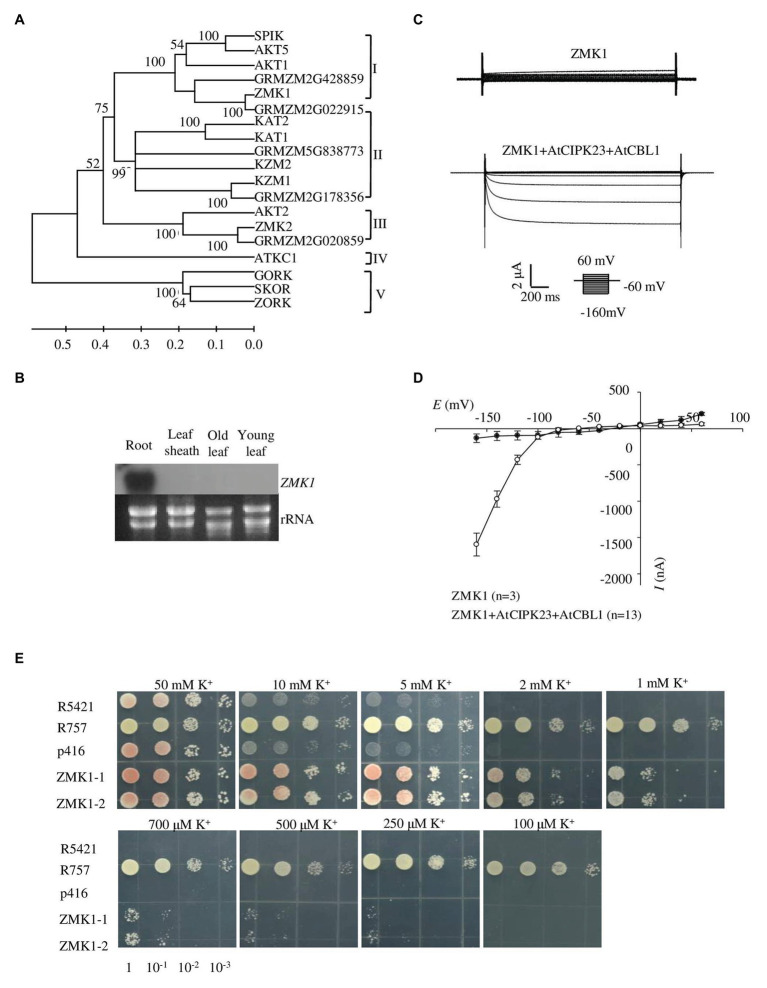
Expression pattern and functional characterization of ZMK1. **(A)** The phylogenetic tree constructed using MEGA4 software based on alignments of all the Shaker-like potassium (K^+^) channels from *Arabidopsis* and maize. The scale bar represents amino substitutions and the numbers on the tree nodes represent bootstraps from 1,000 replicates. **(B)** Northern analysis of *ZMK1* in maize root, leaf sheath, old leaf, and young leaf during V3 stage. The RNA loading in each lane was 20 μg. *rRNA* was used as a quantitative control. **(C)** Representative currents trace upon voltage clamp pulses from 60 to −160 mV in oocytes expressing ZMK1 alone (upper panel) or ZMK1 with AtCIPK23 and AtCBL1 (bottom panel), in 96 mM K^+^ solution at pH 7.2. Values are means ± SE (*n* ≥ 3 oocytes). **(D)** Steady-state current-voltage (*I-V*) relationships for ZMK1 (black circles) alone or ZMK1 with AtCIPK23 and AtCBL1 (open circle) in 96 mM K^+^ solution at pH 7.2.Values are means ± SE (*n* ≥ 3 oocytes). **(E)** Functional characterization of ZMK1 in yeast. ZMK1 complement the K^+^ uptake deficient phenotype of mutant yeast strain R5421 on AP medium containing different K^+^ concentrations. Yeast strain R757 was used as a positive control. The p416 was empty vector.

Several maize genes belonging to the Shaker potassium channel family have been found (Su et al., 2001). To identify the AKT1 homologs in maize, a tblastn search in the Maize sequence^1^ database was conducted by using AKT1 protein sequence. The result showed that about 10 genes belonging to the Shaker-like potassium channel family were present in maize genome. Phylogenetic analysis of these genes with the *Arabidopsis* homologs was performed. *ZMK1*, which was previously reported as the maize homolog of *AKT1*, belongs to the group I of the maize Shaker family (Figure 1A). ZMK1 shows highest amino acid sequence identity with AKT1, and both genes show similar splicing pattern (Supplementary Figures 1, 2A).

Previous studies showed that *ZMK1* was mainly expressed in the coleoptile. It was reported to be in response to auxin and may function in coleoptile growth (Philippar et al., 2006). Although *ZMK1* is considered as an *AKT1* homolog in maize, its physiological roles have not been well characterized. To determine the tissue expression pattern of *ZMK1*, roots, leaf sheaths, old leaves, and young leaves from V3 stage maize seedlings were collected and used for RT-PCR assay. The results indicated that *ZMK1* transcript was predominantly accumulated in root tissues, while rarely detected in other parts (Figure 1B). In *Arabidopsis*, the transcription of *AKT1* is not sensitive to potassium deficiency (Basset et al., 1995), which suggests that AKT1 is mainly regulated at post-transcriptional level. The expression analyses from root indicated that the transcription of *ZMK1* was not significantly changed after low potassium treatment for 12–24 h (Supplementary Figure 2B).

### Identification of ZMK1 Potassium Transport Activity in Yeast and *Xenopus* Oocytes

*Xenopus* oocytes and yeast are important heterologous systems to characterize ion channels and transporters. We investigated the K^+^ channel activity of ZMK1 in *Xenopus* oocytes. When ZMK1 was expressed alone in oocytes, ion currents cannot be recorded. When ZMK1, AtCBL1, and AtCIPK23 were co-expressed in oocytes, the inward potassium currents were recorded (Figure 1C). In oocytes, ZMK1 was activated when the membrane voltage was below −100 mV (Figure 1D). In contrast, AKT1 can be activated when the membrane voltage was below −70 mV, indicating that the activation voltage of ZMK1 is shifted toward negative. The auxotrophic yeast mutant strain R5421 is defective in K^+^ uptake and cannot grow under low-K^+^ conditions. Potassium uptake-deficient yeast mutant R5421 (*trk1∆* and *trk2∆*) fails to grow when K^+^ is less than 5 mM. K^+^ absorption by the yeast strain R757 (*TRK1* and *TRK2*) is not affected by the external environment. We transformed R5421 yeast with *ZMK1*, while used R757 as positive control (Figure 1E). Yeast growth assays were performed on AP medium containing K^+^ concentrations ranging from 0.1 to 50 mM. Yeast strain expressing *ZMK1* can partially restore the growth of yeast strain R5421, allowing R5421 to grow on the AP medium supplemented with 0.25 mM K^+^. Mutant strain R5421 failed to grow under low K^+^ condition (below 2 mM). These results indicated that ZMK1 has K^+^ uptake activity.

### Complementation of *Arabidopsis akt1* Mutant

**Figure 2:**
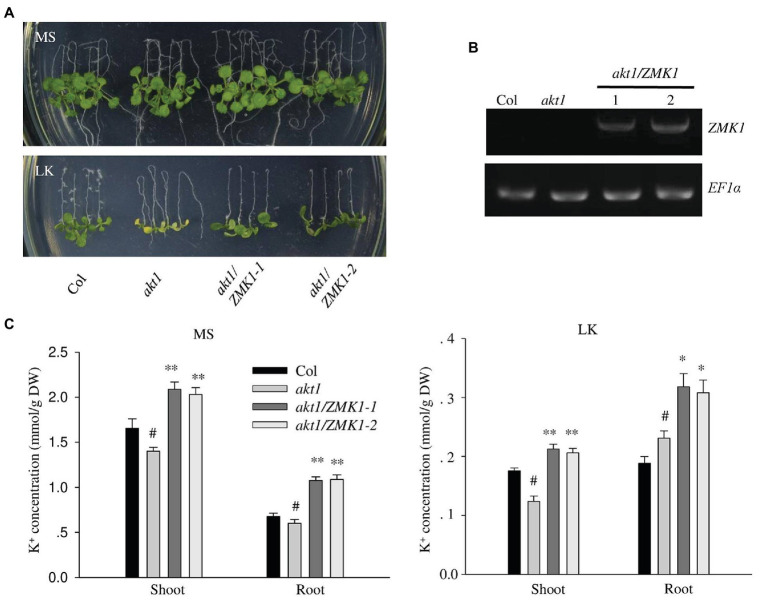
Complementation of *Arabidopsis akt1* mutant by ZMK1. **(A)** Plant materials grown on Murashige and Skoog (MS) medium for 4 days were transferred to MS or LK medium for another 7 days. **(B)** Detection of *ZMK1* expression in different plant materials by reverse transcription PCR (RT-PCR). *EF1a* was used as a quantitative control. **(C)** Quantification of K^+^ concentration in root and shoot parts of different plant materials after grown on MS and LK medium for 7 days. The data are presented as means ± SE (*n* ≥ 3), Student’s *t*-test (^∗^*p* < 0.05, ^∗∗^*p* < 0.01) was used to analyze statistical significance and “*#*” represents the control.

In *Arabidopsis*, AKT1 is reported to function at both high and low potassium conditions. To test whether ZMK1 performs similar function in *Arabidopsis*, the CDS of *ZMK1* under control of the ubiquitin promoter was transformed into the *Arabidopsis* mutant *akt1* (Figure 2A). Two *ZMK1* transgenic lines (*akt1*/*ZMK1*-1 and *akt1*/*ZMK1*-2) were obtained, and the expression of *ZMK1* was verified by RT-PCR assay (Figure 2B). After growth on MS medium for 4 days, the seedlings were transferred to MS and LK, respectively, for another 7 days. The *akt1* mutant displayed leaf chlorosis phenotype on LK medium, while the leaves of *akt1*/*ZMK1* transgenic lines remained green (Figure 2A), suggesting that *ZMK1* could complement the LK sensitive phenotype of *akt1* mutant. The leaf chlorophyll contents were determined, and the results showed that there was no significant difference in chlorophyll content of different materials under MS. Under LK conditions, the leaf chlorophyll content of *akt1* was significantly reduced, while the leaf chlorophyll content of *akt1*/*ZMK1* resumed to the wild-type level. These results were consistent with the phenotypic results (Supplementary Figure 3A). We further measured the K^+^ contents of different plants, the results showed that *ZMK1* fully rescued the K^+^ concentration of *akt1* mutant, to a degree even higher than wild-type plants (Figure 2C). These results suggest that ZMK1 could participate in K^+^ uptake and substitute the function of AKT1 in *Arabidopsis*.

### Identification of Upstream Regulation Factors of ZMK1

Together with calcium binding proteins AtCBL1/9, AtCIPK23 can phosphorylate AKT1 to promote K^+^ uptake in *Arabidopsis* under K^+^ deficient conditions (Xu et al., 2006). To explore the regulation mechanism of ZMK1 in maize, we identified the homolog genes of *AtCBL1/9* and *AtCIPK23* in maize. In the Maize sequence^2^ database, 10 ZmCBLs and 42 ZmCBL-interacting protein kinases (ZmCIPKs) were found. Protein sequences of these genes were aligned with all 10 AtCBLs and 26 AtCIPKs in *Arabidopsis* separately. The alignment results were used to build phylogenetic trees (Supplementary Figures 4A,B). The results showed that the closest homologs of *AtCBL1* and *AtCBL9* were GRMZM2G107575 and GRMZM2G015324, designated as *ZmCBL1* and *ZmCBL9*, respectively. The closest sequence of *AtCIPK23* was GRMZM2G013236, designated as *ZmCIPK23*.

**Figure 3:**
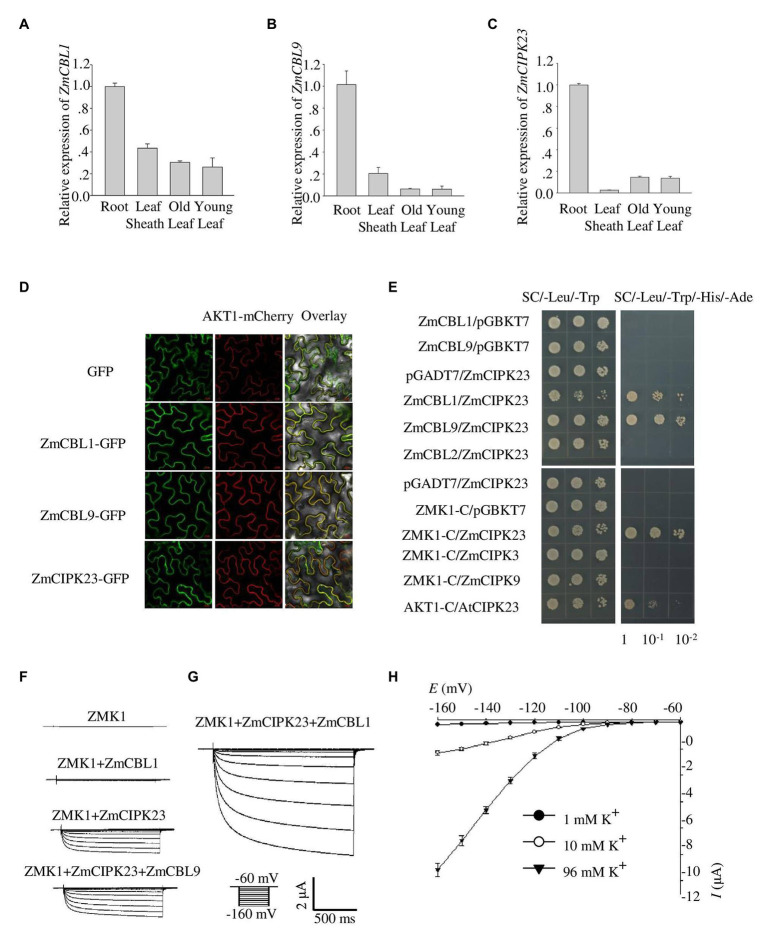
Identification of ZmCBL1, ZmCBL9, and ZmCIPK23. **(A–C)** *ZmCBL1*, *ZmCBL9* and *ZmCIPK23* expression levels in root, leaf sheath, old leaf, and young leaf in V3 stage were quantified by quantitative reverse transcription PCR (RT-qPCR) assay. Values were means ± SE (*n* = 3). *ZmGAPDH* was used as a quantitative control. The data are obtained from three biological replicates. **(D)** Subcellular localization of GFP fusion proteins of ZmCBL1, ZmCBL9, and ZmCIPK23 in *Nicotiana benthamiana*. Bars = 20 μm. AKT1-mCherry was used as the plasma membrane (PM) marker. **(E)** ZmCIPK23 interacted with ZmCBL1, ZmCBL9, and the cytosolic region of ZMK1 in yeast two hybrid assays. The blank pGADT7 and pGBKT7 vector were used as controls. **(F)** From top to bottom, representative currents trace upon voltage clamp pulses from −60 to −160 mV in oocytes expressing ZMK1, ZMK1+ZmCBL1, ZMK1+ZmCIPK23, and ZMK1+ZmCIPK23+ZmCBL9, in 96 mM K^+^ solution at pH 7.2. Values were means ± SE (*n* = 5 oocytes). **(G)** Representative currents trace upon voltage clamp pulses from −60 to −160 mV in oocytes expressing ZMK1+ZmCIPK23+ZmCBL1, in 96 mM K^+^ solution at pH 7.2. Values were means ± SE (*n* = 5 oocytes). **(H)** Steady-state current-voltage (*I-V*) relationships for ZMK1 co-expressed with ZmCIPK23 and ZmCBL1 in 96 mM K^+^ solution at pH 7.2. The external potassium concentration is 1 mM (black circle), 10 mM (open circle), and 96 mM (black triangle). Data are shown as means ± SE (*n* = 5 oocytes).

Quantitative reverse transcription PCR assays indicated that *ZmCBL1*, *ZmCBL9*, and *ZmCIPK23* were highly expressed in maize root, but weak in leaves, indicating that they may regulate ZMK1 in root (Figures 3A–C). The transcription of *ZmCIPK23* showed a strong induction under LK conditions, while the expression of *ZmCBL1* and *ZmCBL9* was not significantly affected (Supplementary Figures 5A–C). Subcellular localization analyses showed that ZmCBL1 and ZmCBL9 were specifically localized on the plasma membrane, while ZmCIPK23 was localized in cytoplasm (Figure 3D).

The fact that *ZmCBL1/9*, *ZmCIPK23*, and *ZMK1* are all preferentially expressed in roots provides a probability that the CBL/CIPK signaling module may also be present in maize. To test protein interaction, yeast two-hybrid assay was performed. The results showed that both ZmCBL1 and ZmCBL9 interacted with ZmCIPK23 (Figure 3E). To testify if ZmCBL1/9 and ZmCIPK23 could modulate the activity of ZMK1, we co-expressed ZMK1 in *Xenopus* oocytes with the combination of ZmCBL1-ZmCIPK23 or ZmCBL9-ZmCIPK23 (Figures 3F–H). The results showed that both combinations could activate the K^+^ transport activity of ZMK1, ZmCBL1-ZmCIPK23 could induce larger currents. The activation voltage of ZMK1 was below −100 mV (Figure 3H). We noted that ZmCIPK23 alone could activate ZMK1, although the K^+^ currents were comparatively small.

### Complementation of *Arabidopsis cipk23* Mutant

**Figure 4:**
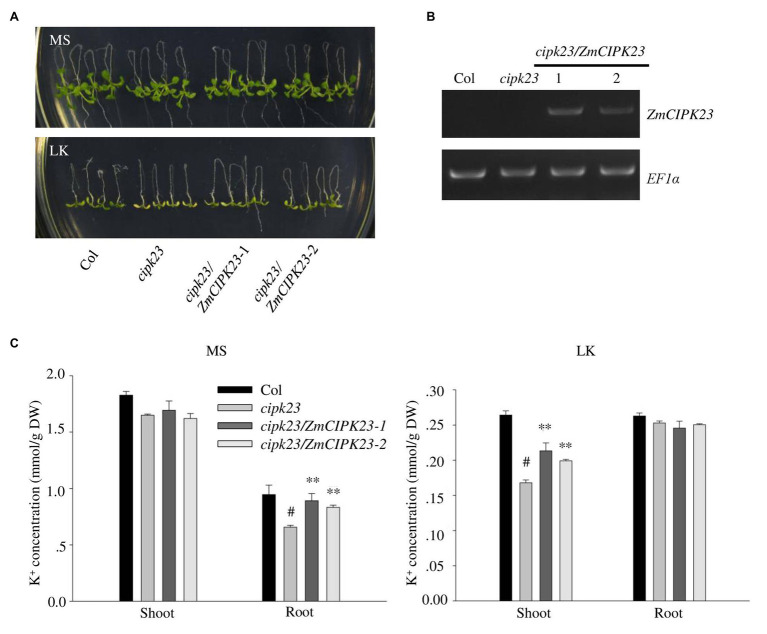
Complementation of *Arabidopsis cipk23* mutant by ZmCIPK23. **(A)** Plant materials grown on MS medium for 4 days were transferred to MS or LK medium for another 7 days. **(B)** Detection of ZmCIPK23 expression in different plant materials by RT-PCR. EF1a was used as a quantitative control. **(C)** Quantification of K^+^ concentration in root and shoot parts of different plant materials after grown on MS and LK medium for 7 days. The data are presented as means ± SE (*n* ≥ 3). Student’s *t*-test (^∗∗^*p* < 0.01) was used to analyze statistical significance and “*#*” represents the control.

The *Arabidopsis cipk23/lks1* mutant was reported to be sensitive to low potassium stress (Xu et al., 2006). To test the function of *ZmCIPK23*, the *ZmCIPK23* CDS was transformed into *Arabidopsis* mutant *cipk23* (Figure 4A). Transgenic lines (*cipk23*/*ZmCIPK23-1* and *cipk23*/*ZmCIPK23-2*) were obtained, and the *ZmCIPK23* expression levels were determined by RT-PCR (Figure 4B). These transgenic lines were used for subsequent phenotype test and K^+^ concentration measurement (Figure 4C). The transgenic lines showed similar phenotype to wild type on LK medium. The leaf chlorophyll contents were consistent with their shoot phenotypes (Supplementary Figure 3B). These results suggest that *ZmCIPK23* has similar function like *AtCIPK23* in *Arabidopsis*.

### ZmCIPK23 Phosphorylates ZMK1

**Figure 5:**
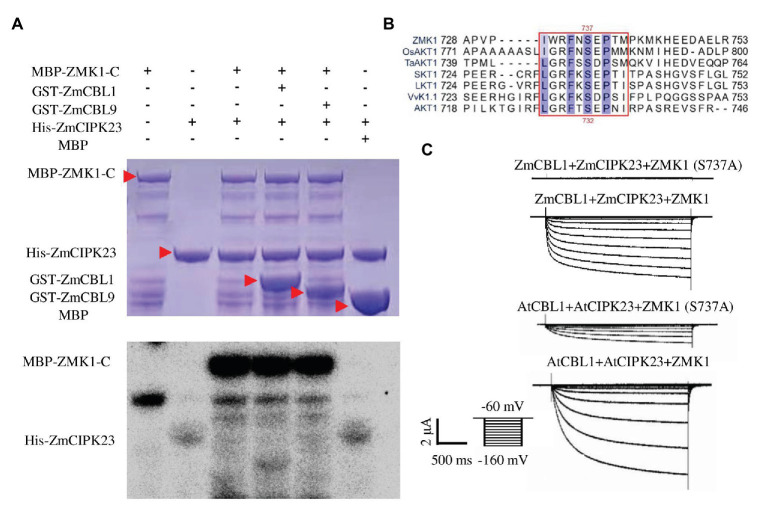
ZmCIPK23 can phosphorylate ZMK1. **(A)** *In vitro* phosphorylation of ZMK1 by ZmCIPK23. ZMK1-C is the cytosolic region of ZMK1 fused with MBP. **(B)** Serine 737 is essential for ZMK1 activation. Sequences were aligned Clustal X software. The amino acids conserved in all species were highlighted, underlining the sequences that fit the conserved phosphorylation motif of CBL-interacting protein kinases (CIPKs). **(C)** From top to bottom, representative currents traces in oocytes expressing ZmCBL1+ZmCIPK23+ZMK1(S737A), ZmCBL1+ZmCIPK23+ZMK1, AtCBL1+AtCIPK23+ZMK1 (S737A), and AtCBL1+AtCIPK23+ZMK1. Values were means ± SE (*n* = 5 oocytes).

ZmCIPK23 was able to interact with the C-terminal intracellular domain of ZMK1 (ZMK1-C, from 303 to 887 amino acids, contain cyclic-nucleotide-binding domain (CNBD), ankyrin domain (ANK1-ANK6), and KHA domain, which do not contain transmembrane domain (S1–S6), and P-loop region; Figure 3E; Supplementary Figure 1). The *in vitro* phosphorylation experiments (Figure 5A) indicated that ZmCIPK23 can phosphorylate ZMK1-C. ZmCBL1, and ZmCBL9 did not significantly enhance the kinase activity of ZmCIPK23. Since both ZMK1 and AKT1 can be phosphorylated by upstream CIPK kinases, we speculated that the phosphorylation sites should be conserved. The amino acid sequences of the intracellular regions of AKT1, ZMK1, OsAKT1, grape VvK1.1, potato SKT1, tomato LKT1, and wheat TaAKT1 were aligned (Figure 5B). The phosphorylation motif of the SOS2 (CIPK24) was reported to be hydrophobic-X-basic-X (2)-S/TX (3)-hydrophobic. The S732 of AKT1 and S737 of ZMK1 fit this motif, thus could be the potential sites for phosphorylation. After mutation of T731A and S732A in AKT1, we detected changes in AKT1 activity in *Xenopus* oocytes when co-expression of AtCBL1 and AtCIPK23. Both T731A and S732A attenuated the inward potassium currents of AKT1. The T731A S732A double mutation further attenuated the inward currents. Therefore, S732 is important for maintaining potassium transport activity of AKT1 (Supplementary Figures 6A–C). The S732 of AKT1 corresponds to the S737 of ZMK1, while the T731 is not conserved. To further investigate the function of S737 in ZMK1, *in vitro* phosphorylation experiments were conducted. Phosphorylation-site mutagenesis verified that S737 is the phosphorylation site on ZMK1 (Supplementary Figure 9). The S737A mutation abolished ZMK1-mediated inward potassium currents in *Xenopus* oocytes when co-expression with ZmCBL1-ZmCIPK23 (Figure 5C), indicating that S737 is critical for the channel activity of ZMK1.

### Physiological Function Analyses of *ZMK1* and *ZmCIPK23* in Maize

**Figure 6:**
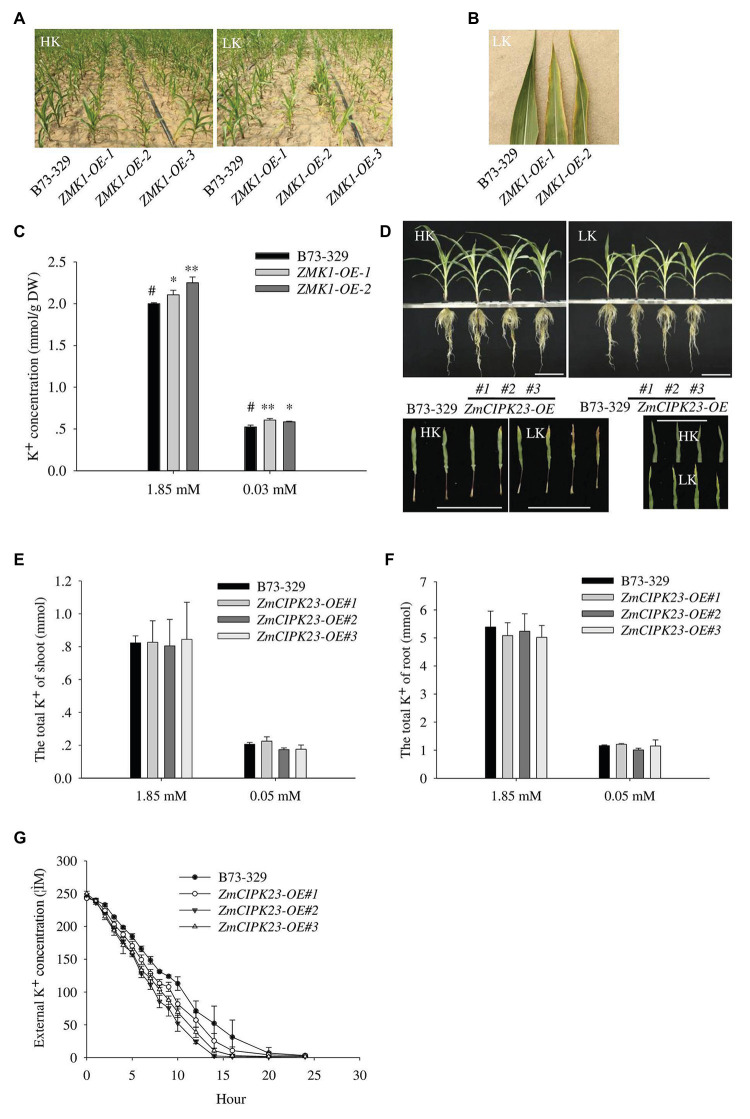
Physiological function analysis of ZMK1 and ZmCIPK23. **(A)** Field phenotypic observation of *ZMK1-OE* in HK or LK fields. **(B)** Leaf phenotype observation in low potassium field. Plants were placed in the order B73-329, *ZMK1-OE-1*, and *ZMK1-OE-2* from left to right. Bars = 8 cm. **(C)** The K^+^ concentration of B73-329 and ZMK1-OE plants (the whole plant) under HK or LK. The data are presented as means ± SE (*n* = 5). Data are shown as means ± SE (*n* = 5). Student’s *t*-test (^∗^*p* < 0.05, ^∗∗^*p* < 0.01) was used to analyze statistical significance and “*#*” represents the control. **(D)** Phenotypic comparison of control plants (B73-329 inbred line), *ZmCIPK23-OE* plants under high K^+^ (HK, 1.85 mmol/L) and low K^+^ (LK, 0.05 mmol/L) conditions (top figure). Comparison the phenotypic of different leaves under high K^+^ and low K^+^ conditions. Plants were placed in the order B73-329, *ZmCIPK23-OE#1*, *ZmCIPK23-OE#2*, and *ZmCIPK23-OE#3* plants (bottom figure). Photographs show maize seedlings grown in HK or LK hydroponic solution for 18 days. Bars = 15 cm. **(E,F)** The total K^+^ of shoot or root in B73-329 or *ZmCIPK23-OE* plants. Data are shown as means ± SE (*n* = 5). Student’s *t*-test (^∗^*p* < 0.05, ^∗∗^*p* < 0.01) was used to analyze statistical significance of differences between genotypes. The data are obtained from five biological replicates. **(G)** Comparison of K^+^ uptake ability between B73-329 and *ZmCIPK23-OE* plants using the K^+^ depletion method. Data are shown as means ± SE (*n* = 3). The data are obtained from three biological replicates.

To characterize the function of *ZMK1*, we constructed the *ZMK1* overexpression lines (*ZMK1-OE*) in maize (B73-329 inbred background; Supplementary Figures 7B,C). We planted *ZMK1-OE* in high potassium or low potassium treated fields. In low-potassium field, *ZMK1-OE* plants exhibited a leaf chlorosis phenotype that is a typical potassium deficient symptom (Figures 6A,B). We validated this phenotype using a hydroponic system. Under high K^+^ (1.85 mM K^+^, HK), there was no significant difference between the *ZMK1-OE* plants and control plants. When plants were treated with low K^+^ (0.03 mM K^+^, LK) for 14 day, the control plants did not show obvious sensitive phenotype (Supplementary Figures 7A,D,E). However, the older leaves of *ZMK1-OE* plants became yellow (Supplementary Figure 7A). Under high or low K^+^ conditions, the K^+^ content of *ZMK1-OE* plants were higher than that of control plants (Figure 6C). Therefore, overexpression of *ZMK1* can increase K^+^ absorption capacity.

We also tested the phenotypes of *ZmCIPK23* overexpressing lines (*ZmCIPK23-OE*). There was no significant difference in biomass between *ZmCIPK23-OE* and control plants under high K^+^ or low K^+^ conditions (Supplementary Figures 8A,B). However, under low K^+^ conditions, the older leaves of *ZmCIPK23-OE* plants became yellow compared with control plants (Figure 6D). There was no difference in total K^+^ content (Figures 6E,F). However, the K^+^-depletion assay indicated that the *ZmCIPK23-OE* plants displayed enhanced K^+^ uptake activity (Figure 6G).

## Discussion

ZMK1 has potassium uptake activity similar to AKT1. However, they showed different channel properties. In *Xenopus* oocytes, ZMK1 can be activated when the membrane voltage was below −100 mV, when the extracellular K^+^ concentration was 96 mM (Figure 3H). Comparatively, AKT1 is activated when the membrane voltage is below −70 mV. These results indicate that the activation voltage of ZMK1 is more negative than AKT1.

AKT1 mediates K^+^ uptake at both high and low K^+^ concentrations. In this study, we found that ZMK1 can mediate inward K^+^ currents in *Xenopus* oocytes at 96, 10, and 1 mM K^+^ conditions. *ZMK1* was able to complement the phenotype and potassium content of *akt1* mutant under MS and LK conditions, indicating that ZMK1 can also function under both high and low (100 μM) potassium conditions.

CIPK proteins are thought to be regulated by upstream CBL proteins, Ca^2+^, and potential protein kinases and phosphatases. Cytoplasm-localized CIPK proteins can be recruited to the membrane by CBLs to regulate substrates, such as ion channels or transporters. It was reported previously that phosphorylation of AtCBL1 is required for *in vivo* activation of AKT1 by AtCBL1-AtCIPK23 complex in oocytes (Hashimoto et al., 2012). AtCIPK23-mediated phosphorylation of AKT1-C is enhanced by AtCBL1 but not by AtCBL1^S201A^. In this study, we found that ZmCIPK23 protein expressed in prokaryotic expression system extract was able to phosphorylate ZMK1 *in vitro* (Figure 5A). We constructed the vector of *ZmCIPK23* removing the auto-inhibitory domain and fused with GST (GST-ZmCIPK23^∆NAF^). *In vitro* phosphorylation results showed that GST-ZmCIPK23^∆NAF^ can phosphorylate ZMK1 (Supplementary Figure 9). Moreover, inward potassium currents can be recorded in *Xenopus* oocytes when co-expression of ZmCIPK23 and ZMK1 (Figure 3F). ZMK1 activity was further enhanced by the addition of ZmCBL1 (Figure 3G). These results indicate that the activity of ZmCIPK23 is not completely dependent on the upstream CBL protein.

Both ZMK1 and ZmCIPK23 could complement the leaf chlorotic and root overgrowth phenotype of *akt1* and *cipk23* mutants, respectively, (Figures 2A, 4A), suggesting that ZMK1 and ZmCIPK23 perform similar function as their *Arabidopsis* counterparts. The primary root growth of mutant *akt1* and *cipk23* on low-potassium medium was faster than that of the wild type. Our previous research (Li et al., 2017) showed that under low potassium conditions, AKT1 can sense and respond to external low-potassium stress, slowing down the vesicle transport process of root cells and starting the PIN1 degradation pathway, leading to the inhibition of the root growth. In *akt1* and *cipk23* mutants, however, such response might have been attenuated, leading to root overgrowth.

In this study, according to sequence alignment of AKT1 homologs, we predicted that S732 on AKT1 and S737 on ZMK1 should be the potential sites phosphorylated by AtCIPK23 or ZmCIPK23 (Supplementary Figures 6A–C). Mutation of these two sites significantly reduced AKT1- and ZMK1-mediated inward potassium currents. *In vitro* phosphorylation assays verified that the S737 is the important phosphorylation site on ZMK1 (Supplementary Figure 9).

Our previous study reported that overexpression of *ZmHAK5* can improve K^+^ uptake capacity in maize (Qin et al., 2019). In this study, we found that overexpressing *ZMK1* increased K^+^ uptake; however, the *ZMK1/ZmCIPK23-OE* plants displayed leaf chlorotic phenotype under low K^+^ conditions, particularly in the old leaves (Figures 6B,D). Since K^+^ is a mobile macronutrient that can be mobilized from older leaves to younger leaves, we speculate that the overexpression of *ZMK1/ZmCIPK23* might affect the distribution of potassium ions in leaves. The ectopic expression of *ZMK1//ZmCIPK23* in shoot with the constitutive promoter may affect K^+^ homeostasis in leaves and cause this sensitive phenotype. Further studies using the tissue-specific promoters to express *ZMK1/ZmCIPK23* may help to solve this problem.

## Data Availability Statement

The datasets generated for this study are available on request to the corresponding authors.

## Ethics Statement

Animal studies were conducted in accordance with the ethical guidelines of Ministry of Agriculture (Beijing, China). All procedures were approved by the Animal Care ethics Committee of China Agricultural University.

## Author Contributions

YW designed the research. WH, YJ, HQ-F, WW, and JK-C conducted the experiments and analyzed the data. YW and HQ-F wrote and revised the article. All authors contributed to the article and approved the submitted version.

### Conflict of Interest

The authors declare that the research was conducted in the absence of any commercial or financial relationships that could be construed as a potential conflict of interest.

## Footnotes

^1^http://www.fao.org/3/ca6746en/ca6746en.pdf

^1^www.maizesequence.org

^2^www.maizesequence.org
