# Supplementary material for: ZMK1 Is Involved in K+ Uptake and Regulated by Protein Kinase ZmCIPK23 in Zea mays
Source: Front Plant Sci. 2021 Mar 3;12:517742. doi: 10.3389/fpls.2021.517742 (PMC7966722; doi:10.3389/fpls.2021.517742)
Supplement: Supplementary file 1 [file Data_Sheet_1.pdf]

Supplement Figure 1

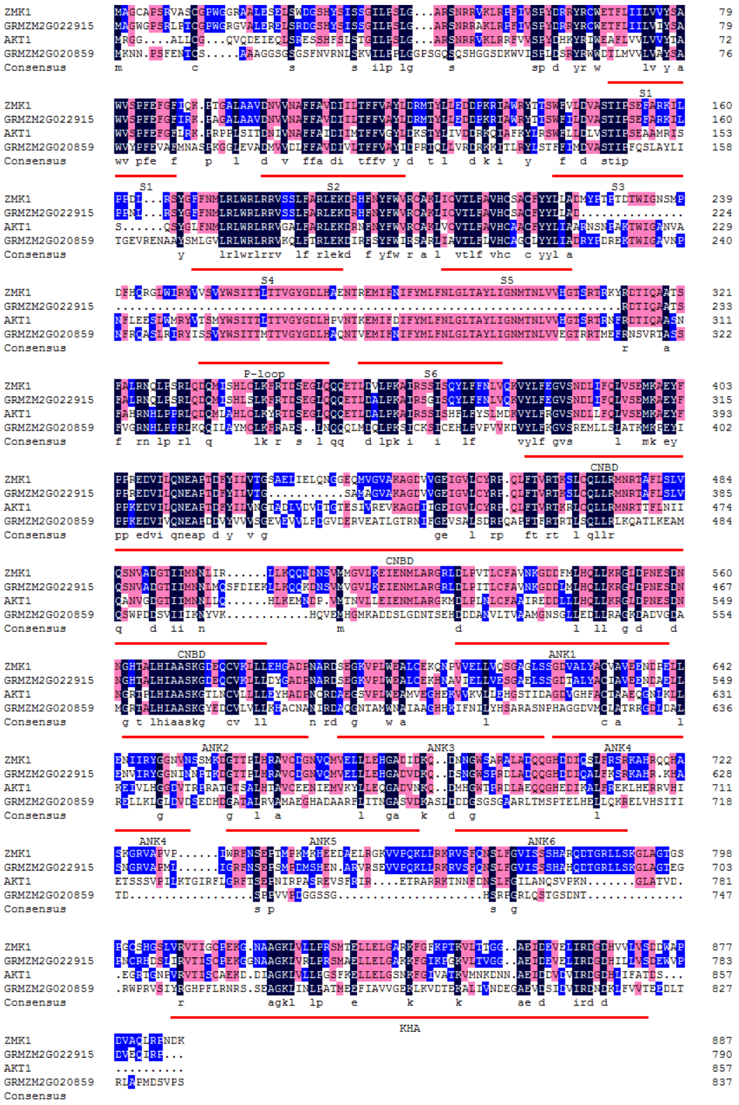

Supplement Figure 1. Alignments of the amino acid sequences of AKT1 and Shaker potassium channels in maize

Alignment was conducted using ClustalW software. Identical residues are in blue/black/pink color. Underlined sequences are transmembrane domain (S1-S6), P-loop region, cyclic-nucleotide-binding domain (CNBD), ankyrin domain(ANK1-ANK6) and KHA domain. Note that GRMZM2G022915 lacks the P-loop region and the S6 transmembrane domain.

Supplement Figure 2

A

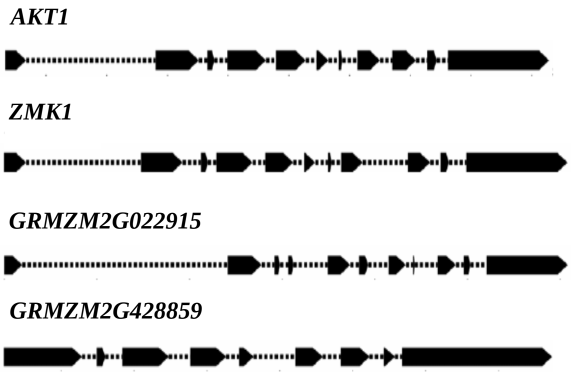

B

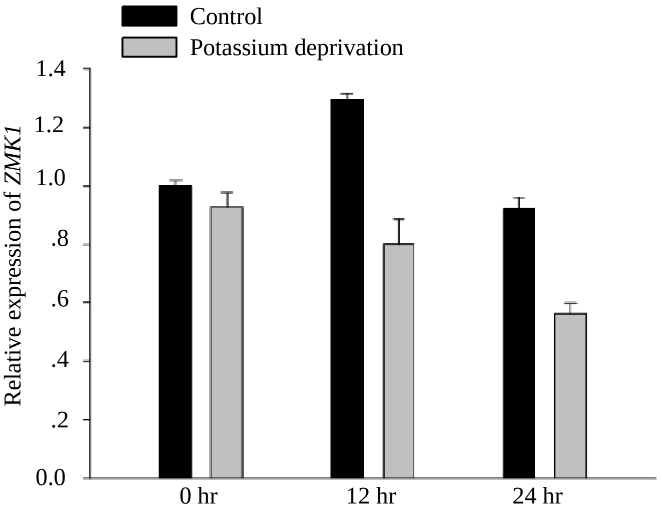

Supplemental Figure 2. Representation intron–exon structure of of AKT1 and Shaker potassium channels in maize and RT-qPCR analysis of *ZMK1* transcript levels in maize in response to K^+ starvation

(A) Representation intron–exon structure of AKT1, ZMK1, GRMZM2G022915 and GRMZM2G428859. The genome sequence and coding sequence were used to draw the gene structure. The exons are indicated by black arrows, and the dot lines represent introns. **(B)** Maize seedlings grown to three leaves stage were transfered to hydroponic solution containing 1 mM or 0 mM K^+ respectively for 12 and 24 hr. The expression of ZMK1 in the root tissue was determined by RT-qPCR assay. *ZmGAPDH* was used as a quantitative control. Values are means ± SE (n=3). Data was obtained from three biological replicates.

Supplemental Figure 3

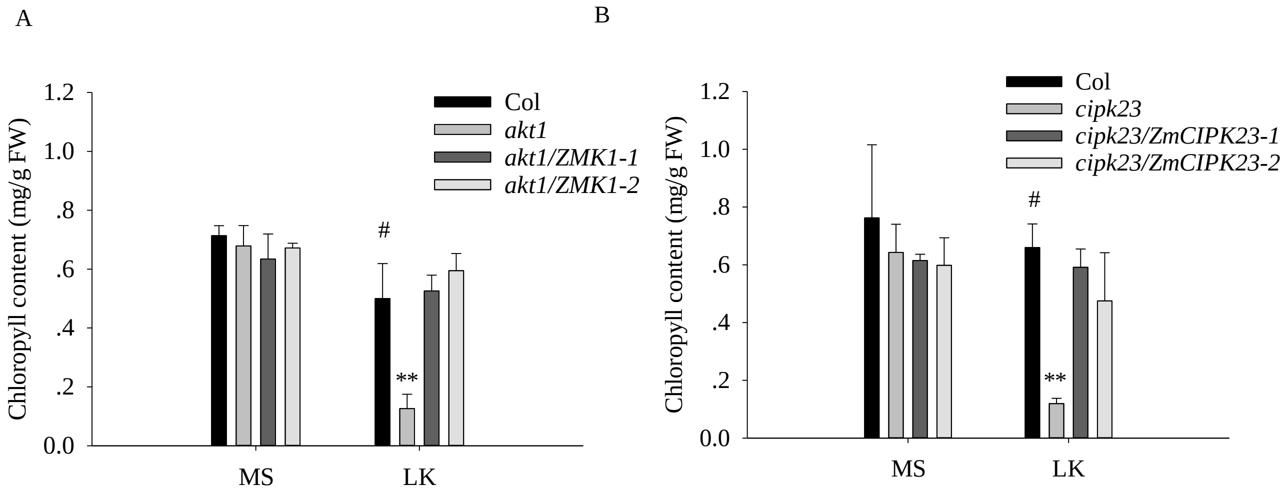

Supplemental Figure 3. Phenotype tests of complementation lines under LK stress

(A) Measure the chlorophyll content in the shoots of *akt1* and *akt1*/ZMK1 under LK stress. Data are shown as means ± SE (n=5). Student's t-test (*P < 0.05, **P < 0.01). **(B)** Measure the chlorophyll content in the shoots of *cipk23* and *cipk23*/ZmCIPK23 under LK stress. Data are shown as means ± SE (n=5). Student's t-test (*P < 0.05, **P < 0.01).

Supplement Figure 4

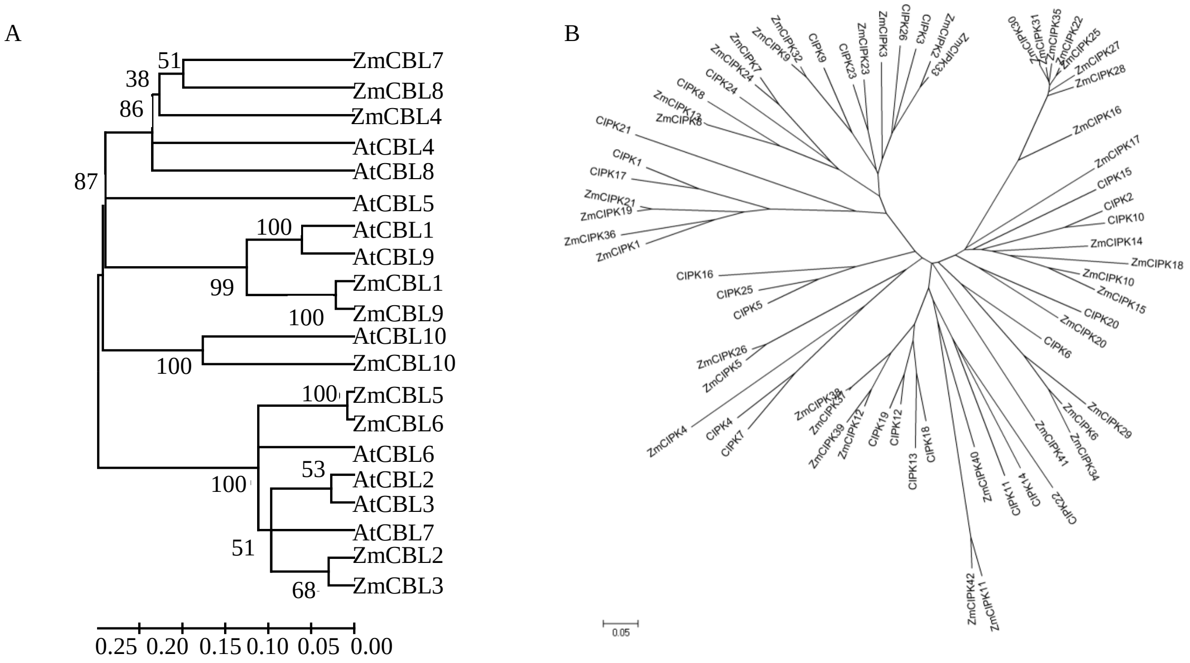

Supplement Figure 4. Phylogenetic relationships of *Arabidopsis* and maize CBLs and CIPKs

(A) Phylogenetic relationships of CBLs in *Arabidopsi*s and maize.

(B) Phylogenetic relationships of CIPKs in *Arabidopsis* and maize.

Supplement Figure 5

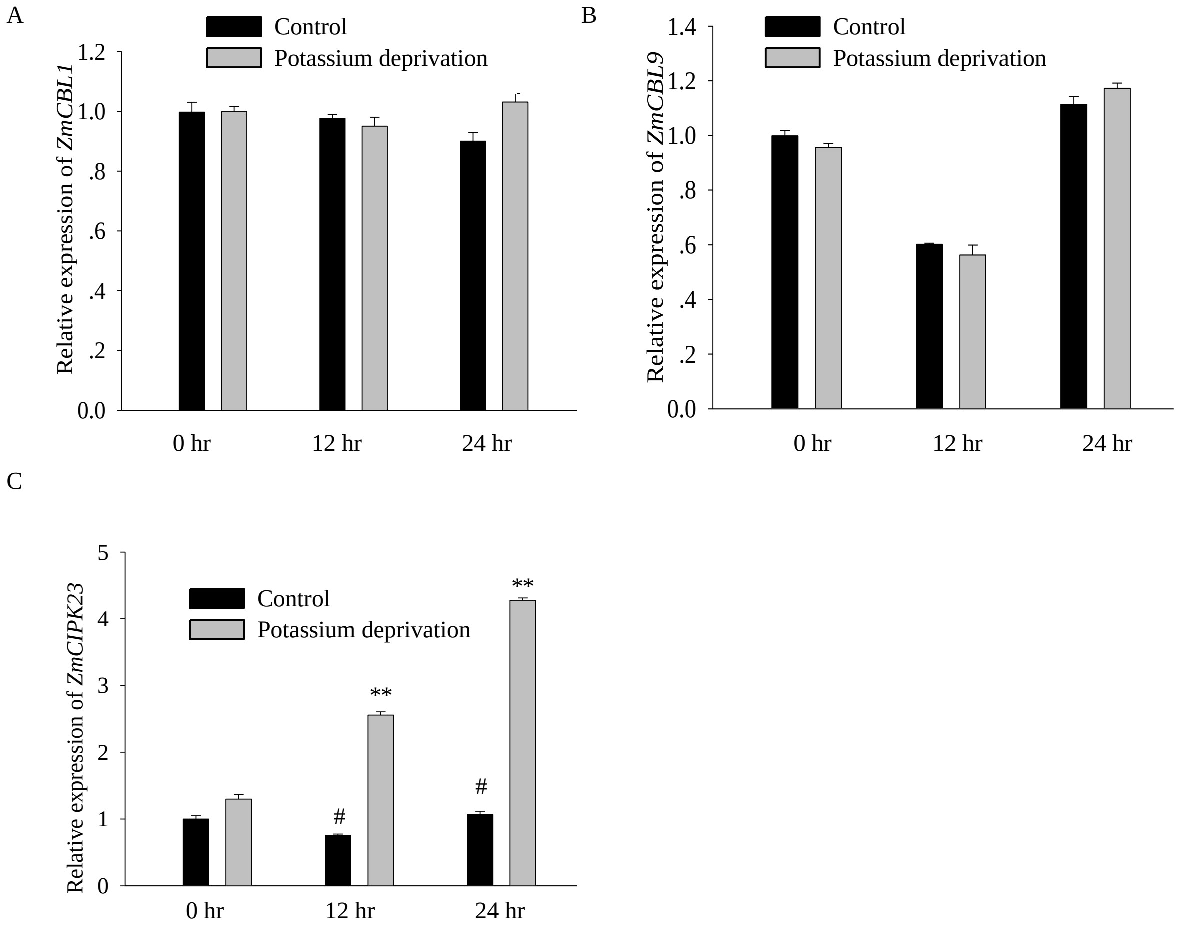

Supplement Figure 5. Effect of potassium deprivation stress on *ZmCBL1/9* and *ZmCIPK23* expression

(A, B, C) Roots were collected from maize seedlings grown to the three leaves stage treated in hydroponic solutions containing 1 mM K^+ or 0 mM K^+ for 12 and 24 hr. Root tissues from three seedlings were collected as one biological replicate. RNA was extracted and the expression levels were quantified by RT-qPCR. *ZmGAPDH* was used as a quantitative control. Values are means $\pm$ SE (n=5). Student's t-test (*P < 0.05, **P < 0.01).

Supplemental Figure 6

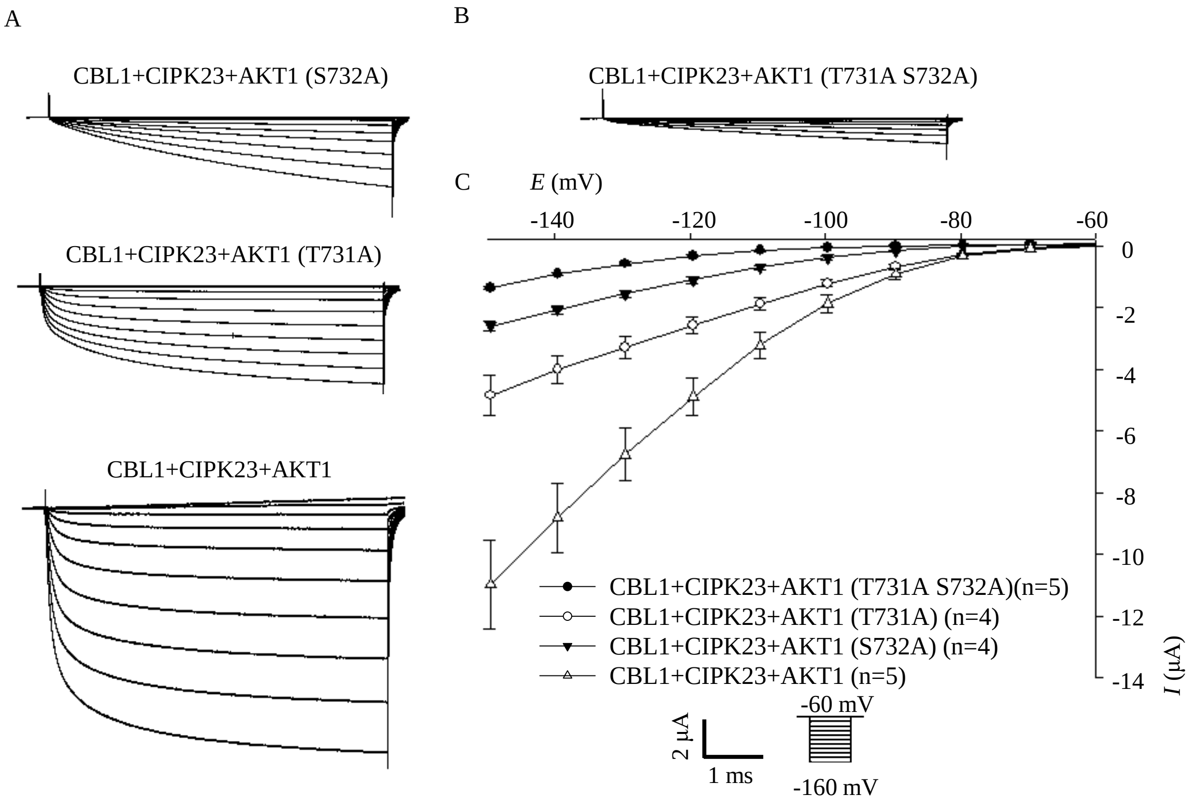

Supplemental Figure 6. Possible phosphorylation sites of AKT1

(A) From top to bottom, representative currents traces upon voltage clamp pulses from -60 mV to -160 mV in oocytes expressing CBL1+CIPK23+AKT1(S732A), CBL1+CIPK23+AKT1(T731A), CBL1+CIPK23+AKT1 in 96 mM K^+ solution at pH 7.2. Values are means ± SE (n=5).

(B) Representative currents traces upon voltage clamp pulses from -60 mV to -160 mV in oocytes expressing CBL1+CIPK23+AKT1(T731A S732A), in 96 mM K^+ solution at pH 7.2. Values are means ± SE (n=5).

(C) Steady-state current-voltage (*I*-*V*) relationships for different version of AKT1 co-expressed with AtCIPK23 and AtCBL1 in 96 mM K^+ solution at pH 7.2. The external potassium concentration is 96 mM. Values are means ± SE (n=5).

Supplement Figure 7

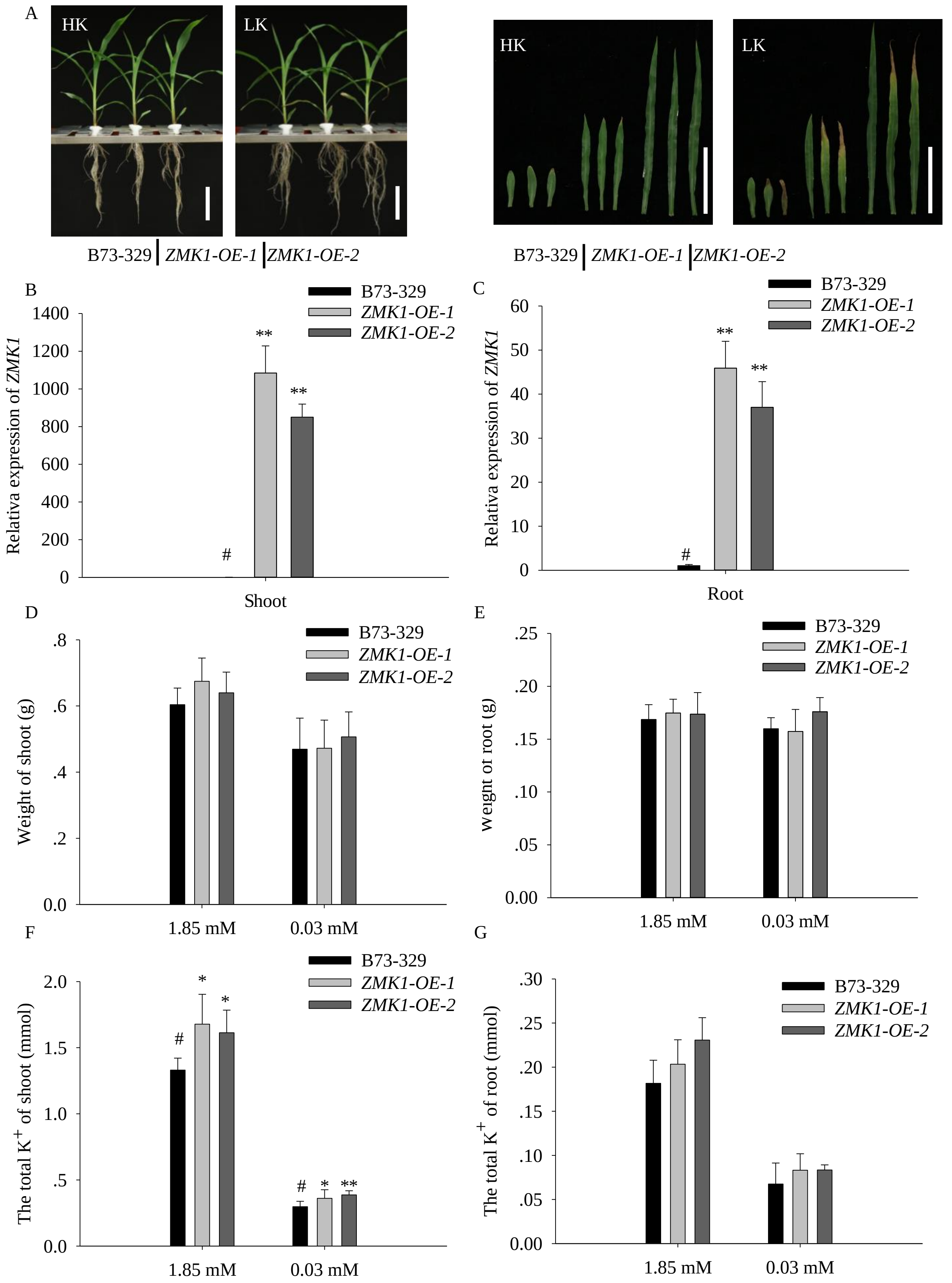

Supplement Figure 7. Physiological Function Analysis of ZMK1

(A) Phenotypes of control plants (B73-329 inbred line) and *ZMK1-OE* plants under high K$^+$ (HK, 1.85 mmol/L) and low K$^+$ (LK, 0.03 mmol/L) conditions. Photographs show maize seedlings grown in HK or LK hydroponic solution for14 d. Bars=8 cm**. (B,C)** Relative expression level of *ZMK1 by root or shoot.*. *ZmUBQ* was used as a quantitative control. Values are means ± SE (n≥3). **(D,E)** The shoot or root dry weight of B73-329 and *ZMK1-OE*. Values are means ± SE (n≥3). **(F,G)** The K$^+$ concentration of B73-329 and *ZMK1-OE* shoot or root under HK or LK. The data are presented as means ± SE (n=5).

Supplement Figure 8

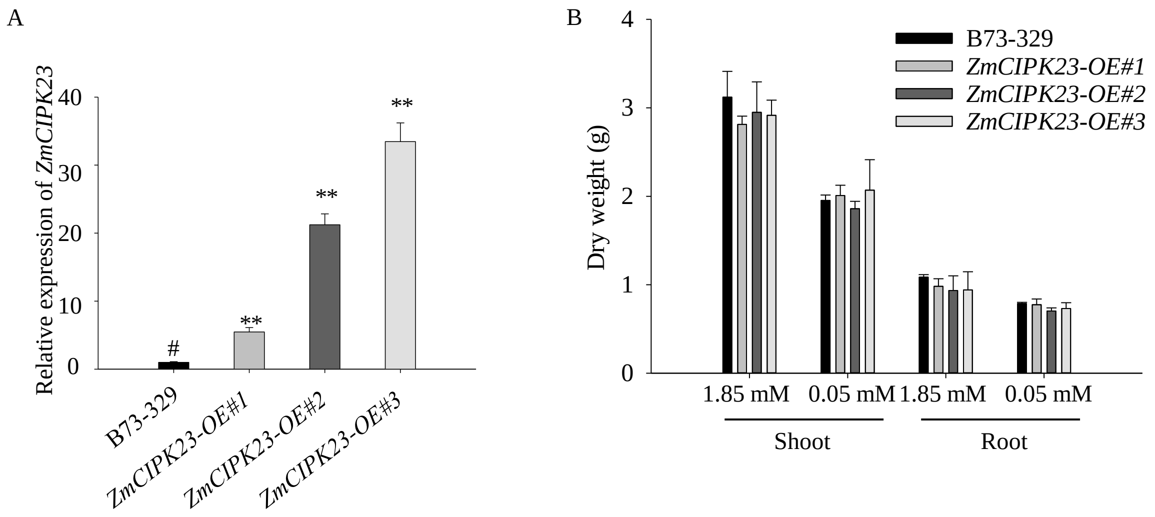

Supplement Figure 8. Phenotypes of *ZmCIPK23-OE* plants

(A) Relative expression level of *ZmCIPK23*. *ZmUBQ* was used as a quantitative control. Values are means ± SE ($n \geq 3$).
(B) The dry weight of *ZmCIPK23-OE* under high K^+ (HK, 1.85 mmol/L) or low K^+ (LK, 0.05 mmol/L). Hydroponic solution for 18 d. Values are means ± SE (n=5). Student's t-test (*$P < 0.05$, **$P < 0.01$).

Supplement Figure 9

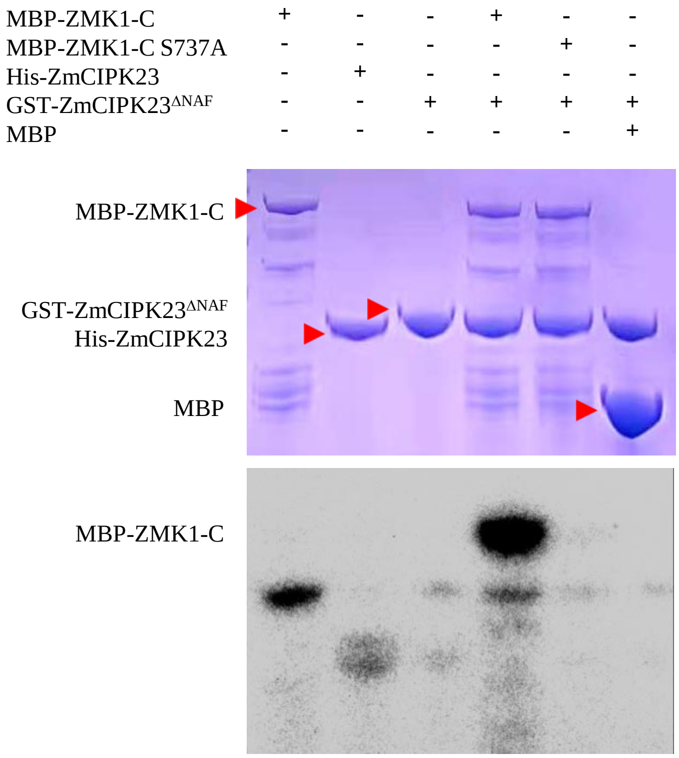

Supplement Figure 9. In vitro phosphorylation of different version of ZMK1 by ZmCIPK23
Kinase assay was initiated by adding radiolabled ATP to the protein mixture as indicate. The reaction was performed at 30°C for 30 min. Top panel: SDS-PAGE gel with Coomassie blue-stained ZmCIPK23 and different version of ZMK1-C proteins; Bottom panel: autoradiograph showing phosphorylation of ZMK1 cytosolic protein (red arrow) by ZmCIPK23.
