# Supplementary material for: ZMK1 Is Involved in K+ Uptake and Regulated by Protein Kinase ZmCIPK23 in Zea mays
Source: Front Plant Sci. 2021 Mar 3;12:517742. doi: 10.3389/fpls.2021.517742 (PMC7966722; doi:10.3389/fpls.2021.517742)
Supplement: Supplementary file 2 [file Data_Sheet_2.pdf]

Supplemental Data Table 1

Primer Name	Primer sequence (5'–3')
**Primers for qPCR**	
ZmCBL1-QC-F	TTCCAGCTTTCTCTGGCAAT
ZmCBL1-QC-R	GGAGTGAATTGGAGGGCATA
ZmCBL9-QC-F	CGTCTCATCTCCCTTTTTCG
ZmCBL9-QC-R	CCCTTCGACGCTTCTTCTAC
ZmCIPK23-QC-F	ATGAATCCGGGGAGCCAGCACA
ZmCIPK23-QC-R	TGCTTCTCGAAGAGCGTCCCGA
ZMK1-QC-F	TCGCGGAAAGCGCATAGGCA
ZMK1-QC-R	GCGGAACAACTTTGCCGCGT
ZmGAPDH-QC-F	CCCCTGCTTCTCATGGATGGTTGC
ZmGAPDH-QC-R	AGGCACGCGCCATCCACATTT
**Primers for Nothern Blot**	
Probe-ZmCIPK23-F	ACTATTGCAGAGCTCATTAAC
Probe-ZmCIPK23-R	GATGCCGCTCTCCGGCTTCC
Probe-ZMK1-F	CTTCTAGAGCACGGAGCTGATC
Probe-ZMK1-R	GTGGGACGAAGAGATGACACCG
**Primers for Yeast assay**	
pGADT7-ZmCBL1-F	TTACCCGGGTATGGGGTGCTTCCATTCCAC
pGADT7-ZmCBL1-R	TTACTCGAGTCACGTGACGAGATCGTCGA
pGADT7-ZmCBL2-F	GGAATTCCATATG ATGGTGCAGTGCCT
pGADT7-ZmCBL2-R	CGGGATCC TCAGTTATCA TCAACTTGGG
pGADT7-ZmCBL9-F	TTACCCGGGTATGGGATGCTTCCATTCCAC
pGADT7-ZmCBL9-R	TTACTCGAGTCACGTGACGAGATCATCGA
pGBKT7-ZmCIPK3-F	GGAATTCCATATG ATGTATCGGG CTAAGAGGGC
pGBKT7-ZmCIPK3-R	CGGGATCC TCATGCTGCC GCG
pGBKT7-ZmCIPK9-F	GGAATTCCATATG ATGGCGGAGC CCG
pGBKT7-ZmCIPK9-R	CGGGATCC TCACTTCGTC TGTTTCATACTTG
pGBKT7-ZmCIPK23-F	TTACCCGGGGATGAGCGCATCCGTGGGCCG
pGBKT7-ZmCIPK23-R	TTAGTCGACTCACGGGGACCTCCTTTGCC
pGADT7-cZMK1-F	TTACCCGGGTCATGGCACCAGCCGTACTCG

pGADT7-cZMK1-R	TTACTCGAGTCACTTGTCGTTCGGTCTTA

**Primers for Yeast assay**	
p416-ZMK1-F	CTCGAGATGGCAGGGTGCGCACCCTC
p416-ZMK1-R	AAGCTTTCACTTGTCGTTCGGTCTTA
p413- ZmCBL1-F	CTCGAGATGGGGTGCTTCCATTCCAT
p413- ZmCBL1-R	AAGCTTTCACGTGACGAGATCGTC
p424- ZmCIPK23-F	CTCGAGATGAGCGCATCCGTGGGC
p424- ZmCIPK23-R	AAGCTTTCACGGGGACCTCCTTTGCC
**Primers used in transgenic construction**	
ZmCIPK23-COM-F	ATGAGCGCATCCGTGGGCCG
ZmCIPK23-COM-R	TCACGGGGACCTCCTTTGCC
ZMK1-COM-F	ATGGCAGGGTGCGCACCCTC
ZMK1-COM-R	TCACTTGTCGTTCGGTCTTAATTGTGCAA
ZMK1-F	ACAAGCAGGACAACAATGGCT
ZMK1-R	CAGCGTCTTCTTCATGTTTCATC
zmk1 cas9-F	GTGCAGTGCAATTTATCACTGTTT
zmk1 cas9-R	TGGTCGCTGCCTGAATTGTA
**Primers for oocytes assay**	
pGEMHE-ZmCBL1-F	TTCCCGGGATGGGGTGCTTCCATTCCACGG
pGEMHE-ZmCBL1-R	TTTCTAGATCACGTGACGAGATCGTCGA
pGEMHE-ZmCBL9-F	TTCCCGGGATGGGATGCTTCCATTCCACGG
pGEMHE-ZmCBL9-R	TTTCTAGATCACGTGACGAGATCATCGA
pGEMHE-ZmCIPK23-F	TTCCCGGGATGAGCGCATCCGTGGGCCGGA
pGEMHE-ZmCIPK23-R	TTTCTAGATCACGGGGACCTCCTTTGCC GC
pGEMHE-ZMK1-F	TTAGATCTATGGCAGGGTGCGCACCCTC
pGEMHE-ZMK1-R	TTGGTACCTCACTTGTCGTTCGGTCTTAATTGTGCAA

Primer Name	Primer sequence (5’–3’)
**Primers for Phosphorylation assay**	
pGEX-4T-1-ZmCBL1-F	CGGGATCC ATGGG GTGCT TCCAT TC
pGEX-4T-1-ZmCBL1-R	CCGCTCGAG TCACGTGACG AGATCGT
pGEX-4T-1-ZmCBL9-F	CGGGATCC ATGGGATGCT TCCATTCC
pGEX-4T-1-ZmCBL9-R	CCGCTCGAG TTACTTGAGG TATGGCAGAG TCATTATCT
pGEX-4T-1-ZmCIPK23 $^{\Delta NAF}$-F	CGGGATCC ATGAGCGCAT CCGT
pGEX-4T-1-ZmCIPK23 $^{\Delta NAF}$-R	CCGCTCGAG TCATCTTTCT TCTCTCCTCT CAACA
pET-30a- ZmCIPK23-F	GCCATGGCTGATATC GGATCC ATGAGCGCAT CCGT
pET-30a- ZmCIPK23-R	GTGGTGGTGGTGGTG CTCGAG CGGGGAC CTCCTTT
pMAL-c5X-cZMK1-F	AGGATTTCACATATGCATGGCACCA GCCGTACTC
pMAL-c5X-cZMK1-R	GAATTCGGATCCGTCGACTCACTTGTCGTTCGGTCT TAATTGT
cZMK1-S737A-F	CGATATGGAGGTTCAACGCCGAACCCACGA TGCCAAA
cZMK1-S737A-R	TTTGGCATCGTGGGTTCGGCGTTGAACCTC CATATCG
